# COVID-19 Depression and Infection Prevention Behavior among College Students: A Health Belief Perspective

**DOI:** 10.3390/healthcare10102104

**Published:** 2022-10-20

**Authors:** Yeong-Mi Jang, Jong-Uk Park, Nam-Yi Kim

**Affiliations:** 1 Department of Nursing, Daejeon Institute of Science and Technology, Daejeon 35408, Korea; 2 Department of Medical Artificial Intelligent, Konyang University, Daejeon 35365, Korea; 3 Department of Nursing, Konyang University, Daejeon 35365, Korea

**Keywords:** COVID-19, depression, health behavior, health belief, perception, students

## Abstract

The COVID-19 pandemic has resulted in an increase in depression among college students due to anxiety and fear of infection. Nonetheless, COVID-19 infection prevention measures should be actively implemented. In this study, the mediating effect of health belief on the relationship between depression and infection prevention behavior was investigated. A survey of 220 South Korean college students was conducted. Depression was found to be the independent variable, health belief the mediating variable, and infection prevention behavior the dependent variable. The model fit index according to confirmatory factor analysis was found to be suitable. Depression among college students was not directly related to COVID-19 infection prevention behavior; however, depression was confirmed to be related to infection prevention behavior via the mediation of health belief. Arbitration measures, focusing on perceived severity and susceptibility during health belief, are required.

## 1. Introduction

The COVID-19 pandemic, which began in 2019, has caused a global health crisis. Governments all over the world have advised their citizens to practice precautionary, basic infection prevention measures, such as social distancing and personal hygiene [1]. Depending on the situation, the South Korean government issued four levels of concrete infection prevention guidelines, including the use of face mask, hand hygiene, private gathering restrictions, and restrictions on operating hours of public facilities. Although these social restrictions were effective in suppressing the transmission of the virus [2], they have been found to have negatively affect people’s psychosocial health [3].

Depression is a major mental health problem that can lead to disabling psychiatric disorders [4]. Long-term (chronic) depression is becoming more common among people as a result of increased exposure to persistent epidemics and natural disasters [5]. Owing to the considerable limitations imposed on daily life by the COVID-19 pandemic, such as social distancing and isolation, depressive symptoms may worsen significantly [6]. In this context, college students have been identified as being especially vulnerable due to concerns about the course of infection and fear of health and infection risks [7], an uncertain academic situation, transition to remote learning environments [8], and social support [9]. Therefore, it is necessary to fully understand their perceptions about the severity of COVID-19 and susceptibility to infection [10] in order to help them better cope with the situation, comply with public health measures and guidelines, and improve their infection prevention behaviors [10].

Infection prevention behavior can be improved by reinforcing health belief. Health belief model includes the following components: perceived susceptibility (perception about the likelihood of contracting a disease), perceived benefits (perception about the benefits of taking preventive measures for lowering the risk of morbidity and the adverse effects of a disease), perceived severity (perception about the seriousness of the outcome of a disease), perceived barriers (perception about the difficulty in overcoming economic and psychological impediments to engaging in preventive behaviors) [1,11,12].

Depression connected to the pandemic has been reported to be associated with one’s level of awareness of the risk of infection, the severity of infection, and the level of infection prevention behavior (e.g., face mask use) according to cognitive distortions and deficiencies [10,13]. In particular, college students in early adulthood, despite their relatively low risk of COVID-19-related hospitalization and death [14], are reported to be a population group that is highly vulnerable in terms of mental health outcomes [15]; this emphasizes the necessity to concentrate research focus on college students’ COVID-19-related depression. A recent survey of American adults over the age of 18 examined the level of psychological distress during the COVID-19 pandemic and found that the prevalence of stress and depressive symptoms was the highest in the early adult age group (18 to 23 years old) compared to other age groups [15]. A global online survey of 63 countries also found that younger age groups were more vulnerable to symptoms of depression and anxiety [16]. In particular, Asians and Hispanics showed even higher levels of psychological distress related to COVID-19 [17]. Therefore, there is a pressing need to evaluate COVID-19-related mental health problems among Korean college students and provide them with interventions to identify their pandemic-related health belief and stimulate their infection prevention behaviors. This study aims to lay the foundation for interventions to improve college students’ COVID-19 infection prevention behaviors by understanding how depression and health belief affect their infection prevention behavior.

## 2. Materials and Methods

### 2.1. Design

This survey study was conducted to understand the mediating effect of health belief on the relationship between college students’ depression and COVID-19 infection prevention behavior. Depression, health belief, and infection prevention behavior were set as the independent, mediating, and dependent variables, respectively.

### 2.2. Participants and Data Collection

This study was conducted using a self-reported online questionnaire for college students attending South Korean universities. To conduct a structural equation study, the recommended sample size is 10 to 20 times per observation variable, and at least 200 samples are needed to apply the maximum likelihood method [18]. When calculated based on the maximum number of observation variables to be used in this study (*n* = 14), the recommended sample size ranged from 140 to 280.

Data were collected from 10 April to 30 May 2022, through the online community bulletin boards of college students. Recruitment notices for research participation were posted, and students who wished to participate voluntarily could fill out a questionnaire through the link. Considering the dropout rate, data were collected until 220 college students were recruited. After checking the data for validity (exclusion criteria: 10% or more missing values or inaccurate information), all data collected (*n* = 220) were used for analysis.

### 2.3. Ethics Approval

Prior to conducting the study, ethics approval was obtained from the Konyang University Institutional Review Board (approval number: KYU 2022-01-001).

### 2.4. Measuring Instruments

#### 2.4.1. Depression

The Patient Health Questionnaire-9 (PHQ-9) scale, developed by Kroenke (2001) and validated by Park et al., (2010), was used to assess depression [19,20]. The PHQ-9 is a self-reported screening tool intended only to screen for depression and its severity. It consists of nine items corresponding to the nine DSM-IV symptoms of depression. The scale measures the frequency of depressive symptomatology over the last two weeks. Each item is rated on a 4-point Likert scale ranging from 0 to 3 (0 = not at all, 1 = 2 to 3 days or more, 2 = 7 days or more, 3 = every day), with the total score ranging from 0 to 27. Cronbach’s α was 0.81 in Park’s study [20] and 0.91 in the current study.

#### 2.4.2. Health Belief

Health belief was measured based on the four components of the health belief model used in the study by Kim and Jeong (2016): perceived benefits, perceived sensitivity, perceived barriers, and perceived severity [21]; the term “blood borne infection” was replaced with “COVID-19 infection”. Each item was rated on a 5-point Likert scale ranging from 1 to 5. The response scale and the number of items of each component are as follows: perceived benefits (1 = not at all effective, 5 = very effective; 5 items), perceived severity (1 = not at all serious, 5 = very serious; 5 items), perceived sensitivity (1 = none, 5 = very high; 5 items), and perceived barriers (1 = not burdensome at all, 5 = very burdensome; 9 items), totaling 24 items. For perceived benefits, perceived sensitivity, and perceived severity, a higher score indicates a higher level of infection-related health belief about infection. For perceived barriers, however, a higher score indicates a lower level of infection-related health belief. Cronbach’s α was 0.79 in the study by Kim and Jeong [21] and 0.87 in the present study.

#### 2.4.3. Infection Prevention Behavior

To measure COVID-19-related infection prevention behavior, questionnaire items were formulated based on the code of conduct issued and distributed by the Ministry of Health and Welfare and the infection prevention behavior scale developed by Kwak and Kim (2021) [22]. The scale consists of 10 items, including face mask use, hand hygiene, social distancing, and symptom check. Each item was rated on a 5-point Likert scale (1 = strongly disagree, 5 = strongly agree), with a higher score indicating a higher level of infection prevention behavior. Cronbach’s α was 0.81 in the study by Kwak and Kim [22], and 0.84 in the present study.

### 2.5. Data Analysis

The participants’ sociodemographic characteristics were analyzed by frequency, percentage, mean, and standard deviation. The correlations between depression, health belief, and infection prevention behavior were analyzed using Pearson’s correlation coefficient. Model fit was confirmed by the following indices: normed χ2, goodness-of-fit index (GFI), normed fit index (NFI), Tucker–Lewis index (TLI), comparative fit index (CFI), and standardized root mean square residual (SRMR). The mediating effect of health belief on the relationship between depression and infection prevention behavior was identified through covariance structure analysis using the maximum likelihood method. Statistical significance was determined through bootstrapping and the significance level was set to *p* < 0.05.

## 3. Results

### 3.1. Sociodemographic Characteristics of Participants

The participants’ mean age was 21.55 ± 4.59 years. Most of the participants were majoring in health sciences, while the rest were majoring in engineering, humanities, and social sciences. The majority of classes were online with 186 students (84.5%). There were 173 women (78.6%), and 95 participants (43.2%) had contracted the COVID-19 virus. There were 58 people (26.4%) who had a depression cut-off point of 10 or higher (Table 1).

### 3.2. Correlational and Descriptive Statistics

The mean values of the measured variables were as follows: depression 1.37 ± 0.71 (out of 4), health belief 3.55 ± 0.55 (out of 5), and infection prevention behavior 3.61 ± 0.74 (out of 5). The normality of the sample was confirmed based on the skewness and kurtosis values. The absolute value of skewness ranged from 0.05 to 0.86, and the absolute value of kurtosis ranged from 0.17 to 1.58 (Table 1).

Depression was significantly positively correlated with perceived severity (r = 0.21, *p* = 0.002), perceived susceptibility (r = 0.22, *p* = 0.001), and perceived barrier (r = 0.24, *p* < 0.001). Infection prevention behavior had a significant positive correlation with perceived benefit (r = 0.36, *p* < 0.001), perceived severity (r = 0.25, *p* < 0.001), perceived susceptibility (r = 0.22, *p* = 0.001), and perceived barrier (r = 0.16, *p* = 0.019), but no correlation with depression (r = 0.05, *p* = 0.423) (Table 2).

### 3.3. Model Fitness and Path Analysis

The index values for hypothetical model fitness were as follows: χ^2^/DF = 2.64, GFI = 0.90, NFI = 0.89, TLI = 0.90, CFI = 0.92, and SRMR = 0.06. All indices, except for the chi-square test, met the criteria. As the chi-square value appears to reject the model even with a very small difference between the sample and the fit matrix, the model can be considered suitable if other fitness indices are considered [23].

The effect size and significance results for the path in the model are presented in Table 3. The direct pathway from depression to health belief was significant (γ = 0.40, *p* = 0.002), that from depression to infection prevention behavior was not significant (γ = −0.08, *p* = 0.537). The indirect pathway from depression to infection prevention behavior (γ = 0.20, *p* = 0.001) was significant. The direct pathway from health belief to infection prevention behavior was significant (γ = 0.47, *p* = 0.004) (Table 3).

**Figure 1:**
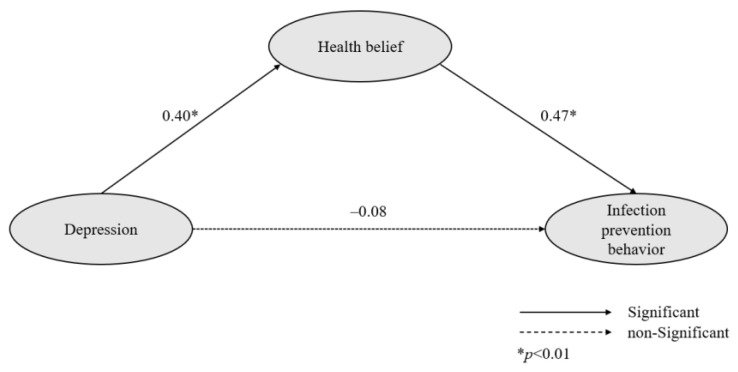
Path diagram of the model.

The direct pathway from depression to infection prevention behavior was not significant, but the indirect pathway was. Thus, it indicates that health belief mediates the relationship between depression and infection prevention behavior (Figure 1).

## 4. Discussion

The results of this study, which was conducted to find a way to stimulate COVID-19 infection prevention behavior among college students, confirmed the mediating effect of health belief on the relationship between depression and infection prevention behavior. Depression among college students was not directly related to their infection prevention behavior but was related to it through the mediating effect of health belief. The significance of this study is twofold: first, it assessed the level of depression among college students during the COVID-19 pandemic; second, it confirmed that psychological factors, such as depression, among college students are related to their health behaviors through the mediation of their perception of health or a disease.

### 4.1. Depression among College Students

In this study, 26.4% of the participants were found to have depressive symptoms, which is significantly higher compared to the pre-COVID-19 prevalence of depression (8.8%) [24], and even higher than the prevalence rate for medical students (21.5%), who are known to have the highest prevalence of depression among all college students [25]. The characteristics of college students make them vulnerable to stress and depression, which may have been exacerbated due to psychological difficulties encountered on a daily basis as a result of the pandemic [7,9]. In particular, anxiety and fear of COVID-19 infection, uncertain academic situation, and abrupt transition to remote learning environments have been reported to be associated with an increase in the prevalence of depression among college students [7,8]. The government should provide accurate information on COVID-19 and clearly present the need for social measures and guidelines for infection prevention. Additionally, it is necessary to help college students to gain a clear understanding of remote learning in their academic process, design online classes that encourage adequate interaction among peers and instructors, and, if necessary, make efforts to offer psychological counseling. At this point in time when the government is moving toward relaxing social distancing measures given a decline in the spread of COVID-19, it is considered all the more important to actively support the use of health interventions for college students.

### 4.2. Mediating Effect of Health Belief

The study’s findings confirmed that health belief mediates the relationship between depression and infection prevention behavior among college students. In particular, the finding that depression is not related to infection prevention behavior directly but only through the mediation of health belief highlights the need for health belief intervention to improve COVID-19 infection prevention behavior. Health belief represents subjective judgments about the likelihood of health outcomes, such as disease, injury, infection, or death. In particular, perceived sensitivity and perceived severity were associated with depression or anxiety during the COVID-19 pandemic [26]. This study also demonstrated that depression is related to perceived severity, sensitivity, and barriers, suggesting that depression among college students affects their health risk perception, which in turn affects their infection prevention behavior to avoid illness. According to the health belief model, increased perceived susceptibility to health problems is associated with an increase in behaviors aimed at reducing the risk of developing health problems. In contrast, people who perceive themselves to be at low risk of contracting a disease are more likely to engage in behaviors that increase their health risk [27]. Perceived severity and perceived sensitivity are correlated with knowledge. In addition, health-related behaviors occur only when perceived benefits are higher than perceived barriers [28]. In this study, the benefits to be gained by engaging in infection prevention behaviors were greater than the barriers (inconvenience, cost, and risk) related to COVID-19. As examined above, health belief is a positive factor for increasing health-promoting behavior.

During pandemics such as the COVID-19, each action of an individual can become a major factor in maintaining the health of the society as a whole. Therefore, it is necessary to consider the social context of college students’ depression and improve their infection prevention behavior by providing them with interventions that improve health belief. In addition, given the impact of psychological factors such as depression on the complex characteristics of human behavior related to health and disease risk [12], more multidimensional research is required.

### 4.3. Limitations

Four limitations of this study are apparent. First, as it was a cross-sectional survey study, additional research needs to be conducted to clarify the causal relationship between the variables. Second, although a descriptive survey was conducted by dividing the sample population into depression and non-depression groups, a group-dependent association between health belief and infection prevention behavior could not be confirmed. Third, care must be taken when interpreting the study results owing to the imbalance in the gender ratio of the study participants. In this study, participants were recruited by uploading a recruitment notice regarding the survey on an online bulletin board. Participants’ personal characteristics influence voluntary participation in online community activities and research [29], and it is thought that the concept of depression and infection prevention behavior has drawn higher interest in female college students. In future studies, gender differences should be considered. Fourth, considering that the correlation between depression, health beliefs, and infection prevention behavior is rather weak, additional research on various factors that can improve infection prevention behavior is needed.

## 5. Conclusions

Depression among college students was not related to their infection prevention behavior directly, but through the mediation of health belief. The COVID-19 pandemic has caused a spike in depression among college students, which underscores the need for health belief interventions to improve their infection prevention behavior; therefore, it is necessary to focus on the severity and sensitivity perceived during health belief. Further research should be conducted to examine in more detail the relationship between health belief and infection prevention behavior by dividing the sample population into depression and non-depression groups.

## Figures and Tables

**Table 1:** Sociodemographic characteristics of participants (*n* = 220).

Variable	Category	*n* (%)	Mean ± SD	Skewness	Kurtosis
Age (years)			21.55 ± 4.59		
Gender	Men	47 (21.4)			
	Women	173 (78.6)			
Grade	Freshmen	42 (19.1)			
	Sophomore	64 (29.1)			
	Junior	54 (24.5)			
	Senior	60 (27.3)			
Major	Health sciences	171 (77.7)			
	Others	49 (22.3)			
Class format	Face to face	34 (15.5)			
	Online	186 (84.5)			
COVID-19 infection experience	Yes	95 (43.2)			
	No	125 (56.8)			
Isolation experience (COVID-19)	Yes	120 (54.5)			
	No	100 (45.5)			
Depression	Yes	58 (26.4)	6.65 ± 6.38	0.86	0.17
(cut-off point = 10)	No	162 (73.6)			
Health belief			3.55 ± 0.55	0.05	0.37
	Perceived benefits		4.20 ± 0.70	−0.77	0.45
	Perceived severity		3.18 ± 0.97	−0.15	−0.35
	Perceived susceptibility		3.74 ± 0.69	−0.70	1.58
	Perceived barriers		3.07 ± 0.92	−0.15	−0.29
Infection prevention behavior			3.61 ± 0.74	−0.24	−0.29

SD = standard deviation.

**Table 2:** Correlation between observed variables.

Variable		DE	PBE	PSE	PSU	PBA	IPB
		R	r				
		(*p*)	(*p*)				
Depression		1					
Health belief	PBE	−0.02	1				
		(0.773)					
	PSE	0.21	0.22	1			
		(0.002)	(0.001)				
	PSU	0.22	0.19	0.29	1		
		(0.001)	(0.006)	(<0.001)			
	PBA	0.24	0.05	0.42	0.30	1	
		(<0.001)	(0.492)	(<0.001)	(<0.001)		
Infection prevention behavior		0.05	0.36	0.25	0.22	0.16	1
		(0.423)	(<0.001)	(<0.001)	0.001	0.019	

DE = depression; IPB = infection prevention behaviors; PBA = perceived barriers; PBE = perceived benefits; PSE = perceived severity; PSU = perceived susceptibility.

**Table 3:** Model fitness and path analysis.

Endogenous Variable	Exogenous Variable	SRW	SE	CR	*p*	Direct β (*p*)	Indirect β (*p*)
Health belief	Depression	0.40	0.04	2.98	0.003	0.40 (0.002)	
Infection prevention behavior	Depression	−0.08	0.09	−0.96	0.337	−0.08 (0.537)	0.20 (0.001)
	Health belief	0.47	0.55	3.08	0.002	0.47 (0.004)	
Goodness-of-fit statistics		χ2/DF(*p*) = 2.64 (<0.001), GFI = 0.90, NFI = 0.89, TLI = 0.90, CFI = 0.92, SRMR = 0.06					

CFI = comparative fit index; CR = composite reliability; DF = degrees of freedom; GFI = goodness-of-fit index; NFI = normed fit index; SE = standard error; SRMR = standardized root mean square residual; SRW = standardized regression weights; TLI = Tucker–Lewis index.

## Data Availability

Data cannot be shared publicly because of restrictions imposed by the Konyang University Institutional Review Board. Data are available from the Konyang University Institutional Data Access/Ethics Committee for researchers who meet the criteria for access to confidential data. Data requests can be addressed to the Konyang University Institutional Review Board (82-42-600-8466, kirb@konyang.ac.kr).
